# Gene model for the ortholog of *rictor* in *Drosophila ananassae*

**DOI:** 10.17912/micropub.biology.000982

**Published:** 2025-04-17

**Authors:** Anne E. Backlund, Brooklin Bain, Kayton Kite, Carter Babbitt, Lindsey J. Long, Judith Leatherman, Brian Schwartz, Chinmay P. Rele, Laura K Reed

**Affiliations:** 1 The University of Alabama, Tuscaloosa, AL USA; 2 Oklahoma Christian University, Edmond, OK USA; 3 University of Northern Colorado, Greeley, CO USA; 4 Columbus State University, Columbus, GA USA; 5 The University of Alabama, Tuscaloosa, AL USA

## Abstract

Gene model for the ortholog of *rapamycin-insensitive companion of Tor* ( *rictor* ) in the May 2011 (Agencourt dana_caf1/DanaCAF1) Genome Assembly (GenBank Accession: GCA_000005115.1 ) of *Drosophila ananassae* . This ortholog was characterized as part of a developing dataset to study the evolution of the Insulin/insulin-like growth factor signaling pathway (IIS) across the genus *Drosophila* using the Genomics Education Partnership gene annotation protocol for Course-based Undergraduate Research Experiences.

**Figure 1. Genomic neighborhood and gene model for rictor in Drosophila ananassae:**
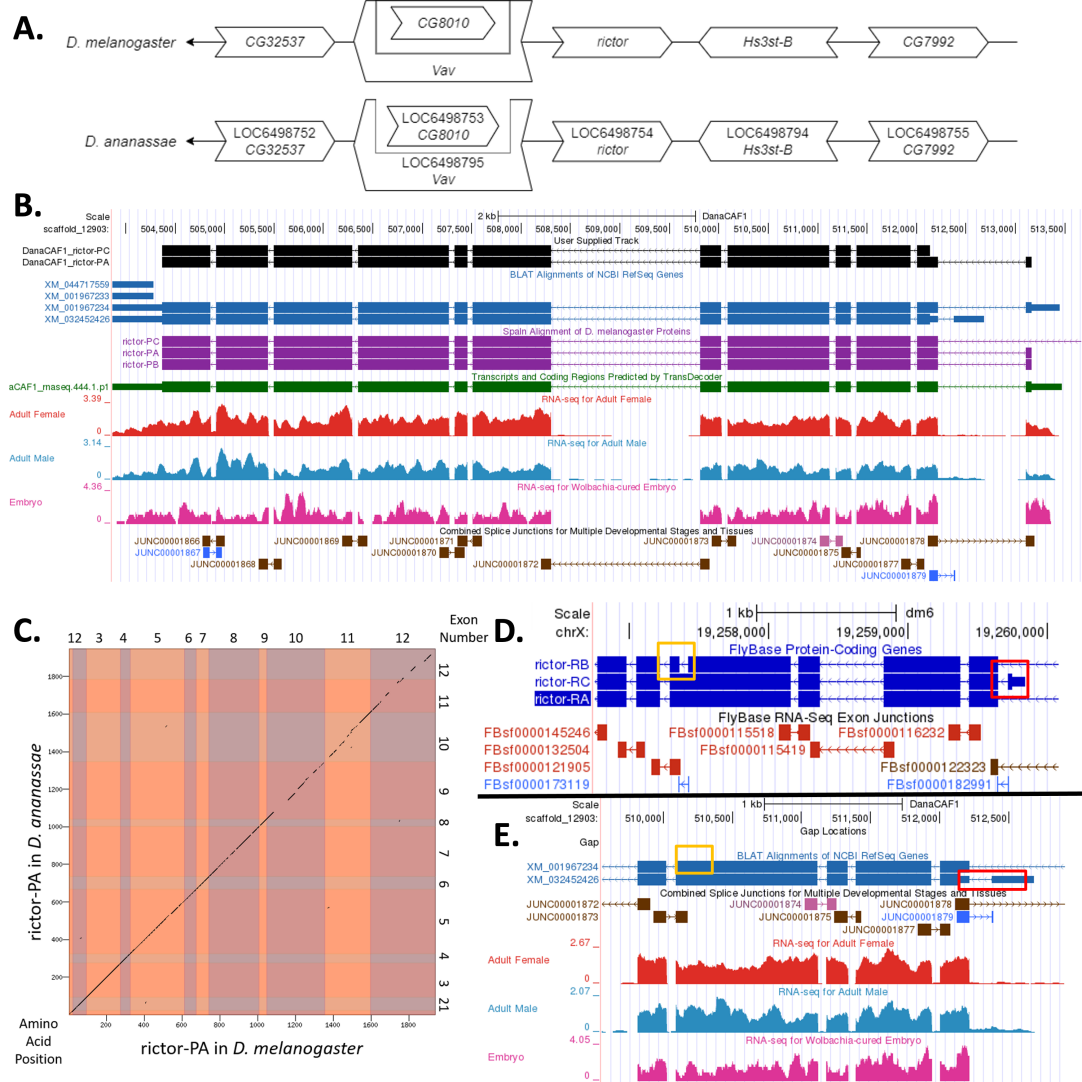
**
(A) Synteny comparison of the genomic neighborhoods for *rictor* in *Drosophila melanogaster* and *D.ananassae* .** Thin underlying arrows indicate the DNA strand within which the reference gene– *rictor* –is located in *D. melanogaster* (top) and *D. ananassae* (bottom). Thin arrows pointing to the left indicate that *rictor* is on the negative (-) strand in *D. ananassae* and *D. melanogaster* . The wide gene arrows pointing in the same direction as *rictor* are also encoded on the negative strand, while the wide gene arrows pointing in the opposite direction of *rictor* are encoded on the positive strand. White gene arrows in *D. ananassae* indicate orthology to the corresponding gene in *D. melanogaster* . Gene symbols given in the *D. ananassae* gene arrows indicate the orthologous gene in *D. melanogaster* , while the locus identifiers are specific to *D. ananassae* . **(B) Gene Model in GEP UCSC Track Data Hub** (Raney et al. 2014). The coding-regions of *rictor* in *D. ananassae* are displayed in the User Supplied Track (black); coding CDSs are depicted by thick rectangles and introns by thin lines with arrows indicating the direction of transcription. Subsequent evidence tracks include BLAT Alignments of NCBI RefSeq Genes (dark blue, alignment of Ref-Seq genes for *D. ananassae* ), Spaln of *D. melanogaster* Proteins (purple, alignment of Ref-Seq proteins from *D. melanogaster* ), Transcripts and Coding Regions Predicted by TransDecoder (dark green), RNA-Seq from Adult Females and Adult Males (red and light blue, respectively; alignment of Illumina RNA-Seq reads from *D. ananassae* ), and Splice Junctions Predicted by regtools using *D. ananassae* RNA-Seq (SRP006203, SRP007906, PRJNA257286, PRJNA388952). Splice junctions shown have a minimum read-depth of 10 with 10-49, 100-499, or 500-999 supporting reads in blue, pink, and brown, respectively. **(C) Dot Plot of rictor-PA in *D. melanogaster* ( *x* -axis) vs. the orthologous peptide in *D. ananassae* ( *y* -axis).** Amino acid number is indicated along the left and bottom; CDS number is indicated along the top and right, and CDSs are also highlighted with alternating colors. Any gaps in the dot plot correspond to portions of the amino acid sequence that lack sequence similarity with *D. melanogaster* . Although the rictor-PA isoform has 12 coding CDSs in both species, the gene structure of *D. ananassae* has diverged relative to that of *D. melanogaster* , with a lost intron near amino acid 600, and a gained intron near amino acid 1800. **(D)** **Special characteristics of the protein model: *rictor* in the *D. melanogaster* GEP UCSC Genome Browser.** The orange box highlights the fifth and sixth CDSs, which make *rictor-RB* unique from *rictor-RA* and *rictor-RC* . The red box highlights the first CDS of *rictor-RC* which makes it unique from the other isoforms. The FlyBase RNA-Seq CDS Junctions track is also displayed. **(E) Special characteristics of the protein model: putative *rictor* ortholog in the *D. ananassae* GEP UCSC Genome Browser.** The orange box highlights the region where, if *rictor-RB* was present, the end of the fifth and the beginning of the sixth CDS would be. We hypothesize that because this fifth and sixth CDS does not appear to exist in the putative ortholog of *rictor* in *D. ananassae* , isoform *rictor-RB* does not exist in this species. The red box highlights the region where the distinctive first CDS of *rictor-RC* would be. Although splice junctions reveal the presence of a transcript in this region, the lack of an in-frame start codon in this CDS led us to hypothesize that the coding region of *rictor-RC* begins in the following CDS in *D. ananassae* . The Combined Splice Junctions for Multiple Developmental Stages and Tissues, and RNA-Seq for Adult Females, Males, and *Wolbachia* -cured Embryos tracks are also displayed.

## Description

**Table:** 

* This article reports a predicted gene model generated by undergraduate work using a structured gene model annotation protocol defined by the Genomics Education Partnership (GEP; thegep.org ) for Course-based Undergraduate Research Experience (CURE). The following information in this box may be repeated in other articles submitted by participants using the same GEP CURE protocol for annotating Drosophila species orthologs of Drosophila melanogaster genes in the insulin signaling pathway. * "In this GEP CURE protocol students use web-based tools to manually annotate genes in non-model *Drosophila* species based on orthology to genes in the well-annotated model organism fruitfly *Drosophila melanogaster* . The GEP uses web-based tools to allow undergraduates to participate in course-based research by generating manual annotations of genes in non-model species (Rele et al., 2023). Computational-based gene predictions in any organism are often improved by careful manual annotation and curation, allowing for more accurate analyses of gene and genome evolution (Mudge and Harrow 2016; Tello-Ruiz et al., 2019). These models of orthologous genes across species, such as the one presented here, then provide a reliable basis for further evolutionary genomic analyses when made available to the scientific community.” (Myers et al., 2024). “The particular gene ortholog described here was characterized as part of a developing dataset to study the evolution of the Insulin/insulin-like growth factor signaling pathway (IIS) across the genus *Drosophila* . The Insulin/insulin-like growth factor signaling pathway (IIS) is a highly conserved signaling pathway in animals and is central to mediating organismal responses to nutrients (Hietakangas and Cohen 2009; Grewal 2009).” (Myers et al., 2024). “ *D* . *ananassae* (NCBI:txid7217) is part of the *melanogaster* species group within the subgenus *Sophophora* of the genus *Drosophila* (Sturtevant 1939; Bock and Wheeler 1972). It was first described by Doeschall (1858). *D. ananassae* is circumtropical (Markow and O'Grady 2005; https://www.taxodros.uzh.ch , accessed 1 Feb 2023), and often associated with human settlement (Singh 2010). It has been extensively studied as a model for its cytogenetic and genetic characteristics, and in experimental evolution (Kikkawa 1938; Singh and Yadav 2015).” (Lawson et al., 2024).

We propose a gene model for the *D. ananassae* ortholog of the *D. melanogaster* *rapamycin-insensitive companion of Tor* ( *rictor* ) gene. The genomic region of the ortholog corresponds to the uncharacterized protein LOC6498754 (RefSeq accession XP_001967270.1 ) in the May 2011 (Agencourt dana_caf1/DanaCAF1) Genome Assembly of *D. ananassae* ( GCA_000005115.1 ; *Drosophila* 12 Genomes Consortium et al., 2007). This model is based on RNA-Seq data from *D. ananassae* ( SRP006203 , SRP007906 ; PRJNA257286 , PRJNA388952 ; Graveley et al., 2011) and *rictor* in *D. melanogaster* using FlyBase release FB2023_02 ( GCA_000001215.4 ; Larkin et al., 2021; Gramates et al., 2022; Jenkins et al., 2022).

The *rictor* gene ( *rapamycin-insensitive companion of Tor* ) encodes a protein that is an essential component of the TOR Complex 2 (TORC2), which regulates actin polymerization, cell growth, heat stress response, and mitochondrial function (Sarbassov et al., 2004; Wang et al., 2012; Jevtov et al., 2015; Liu et al., 2022). *rictor* is frequently misexpressed in human cancers, and its expression is associated with poor survival (Gkountakos et al., 2018). TORC2 directly phosphorylates the Akt “hydrophobic motif,” a phosphorylation event necessary for high levels of Akt signaling typically associated with tissue hyperplasia (Sarbassov et al., 2005; Hietakangas and Cohen 2007). Other targets of TORC2 phosphorylation include trc in *Drosophila* , and PKCalpha and SGK1 in mammals (Koike-Kumagai et al., 2009; Guertin et al., 2006; Garcia-Martinez and Alessi 2008). *Drosophila rictor* mutants are viable and fertile and display mild developmental delay, sensory neuron dendritic tiling defects, and increased synaptic growth at neuromuscular junctions (Hietakangas and Cohen 2007; Koike-Kumagai et al., 2009; Natarajan et al., 2013).

***Synteny***

The reference gene, *rictor ,* occurs on chromosome X in *D. melanogaster* and is flanked upstream by *CG32537* and *Vav guanine nucleotide exchange factor* ( *Vav* ) *. Vav* nests *CG8010* . *rictor* is flanked downstream by *Heparan sulfate 3-O sulfotransferase-B* ( *Hs3st-B* ) and *CG7992* . The *tblastn* search of *D. melanogaster* rictor-PA (query) against the *D. ananassae* ( GCA_000005115.1 ) Genome Assembly (database) placed the putative ortholog of *rictor* within scaffold_12903 at locus LOC6498754 (XP_ 001967270.1)— with an E-value of 5e-04 and a percent identity of 90.48%. Furthermore, the putative ortholog is flanked upstream by LOC6498752 (XP_ 001967273.1) and LOC6498795 (XP_ 032308091.1), which correspond to *CG32537* and *Vav* in *D. melanogaster* (E-value: 5e-103 and 0.0; percent identity: 59.60% and 88.17%, respectively, as determined by *blastp* ) ( Figure 1A, Altschul et al., 1990). The ortholog of *Vav* nests LOC6498753 ( XP_001967272.1 ), which corresponds to *CG8010* in *D. melanogaster* (E-value: 6e-167, percent identity: 66.19%). The putative ortholog of *rictor* is flanked downstream by LOC6498794 ( XP_044573494.1 ) and LOC6498755 ( XP_001967268.1 ), which correspond to *Hs3st-B* and *CG7992* in *D. melanogaster* (E-value: 0.0 and 0.0; percent identity: 91.32% and 84.17%, respectively, as determined by *blastp* ). The putative ortholog assignment for *rictor* in *D. ananassae* is supported by the following evidence: synteny of the genomic neighborhood is conserved and supported by strong BLAST results. In addition, the *tblastn* search of the *D. melanogaster* protein sequence against the *D. ananassae* Genome Assembly gave a very low E-value and a high percent identity, which makes us confident that this is the location of the putative ortholog of *rictor* in *D. ananassae* .

***Protein Model***

*rictor* in *D. melanogaster* has three mRNA isoforms: *rictor-RA* , *rictor-RB* , and *rictor-RC* . However, in *D. ananassae* it appears there are only two mRNA isoforms: *rictor-RA* and *rictor-RC* ( Figure 1B ). In *D. melanogaster* , *rictor-RA* and *rictor-RC* each contain twelve CDSs, while *rictor-RB* has thirteen. Although *rictor-RA* in *D. ananassae* also has twelve CDSs, the gene structure has changed relative to that of *D. melanogaster,* with one lost intron near amino acid 600 (between CDSs five and six in *D.* *melanogaster* ), and one gained intron near amino acid 1800 (within CDS twelve of *D. melanogaster* ) ( Figure 1C ). Also, in *D. ananassae* it appears there are only eleven CDSs in *rictor-RC* . (For more information about these idiosyncrasies, see “Special characteristics of the protein model” below). The sequence of rictor-PA in *D. ananassae* has 78.95% identity (E-value: 0.0) with the protein-coding isoform rictor-PA in *D. melanogaster* , as determined by *blastp* ( Figure 1C ). Coordinates of this curated gene model are stored by NCBI at GenBank/BankIt (accessions BK064528 and BK064529 ). These data are also archived in the CaltechDATA repository (see “Extended Data” section below).

***Special characteristics of the protein model***

**The putative ortholog of *rictor-RB* does not appear to exist in *D. ananassae***

In *D. melanogaster* , there are three isoforms of rictor: *rictor-RA, rictor-RB* , and *rictor-RC* ( Figure 1D ). However, in *D. ananassae* there appears to only be two isoforms ( Figure 1E ). We determined that one of the isoforms present in *D. ananassae* is *rictor-RA* because it matches the CDS number and structure of the ortholog in *D. melanogaster* . We also determined that the other isoform present in *D. ananassae* is most likely *rictor-RC* . The distinctive fifth and sixth CDSs that make *rictor-RB* unique in *D. melanogaster* were not present in *D. anaassae* (orange boxes in Figure 1D and 1E), which led us to our conclusion. The presence of the fifth and sixth CDSs in *rictor-RB* of *D. melanogaster* are supported by the presence of splice junction FBsf0000173119, which had a score of 29 ( Figure 1D ). In *D. ananassae* , there are no splice junctions present in this region, and the BLAT alignment and RNA-Seq are continuous. Therefore, we hypothesize that the only isoforms of *rictor* in *D. ananassae* are *rictor-RA* and *rictor-RC* .

**The putative ortholog of *rictor-RC* appears to be missing the first CDS**

There were also differences in the putative ortholog of *rictor-RC* in *D. ananassae* and the corresponding isoform in *D. melanogaster* . In *D. melanogaster* , *rictor-RC* has twelve CDSs and has a different first CDS than the other isoforms (red box, Figure 1D ). In *D. ananassae* , the presence of a rare transcript for this alternative first CDS is revealed by the splice junction data (JUNC00001879), but no start codon is present in the alternative CDS ( Figure 1E ). Thus, as predicted in the BLAT Alignment of NCBI RefSeq Genes, we propose that the *rictor-RC* isoform is missing the first CDS relative to the *D. melanogaster* ortholog, and an alternative start codon in the following CDS is used.

## Methods

Detailed methods including algorithms, database versions, and citations for the complete annotation process can be found in Rele et al. (2023). Briefly, students use the GEP instance of the UCSC Genome Browser v.435 ( https://gander.wustl.edu ; Kent WJ et al., 2002; Navarro Gonzalez et al., 2021) to examine the genomic neighborhood of their reference IIS gene in the *D. melanogaster* genome assembly (Aug. 2014; BDGP Release 6 + ISO1 MT/dm6). Students then retrieve the protein sequence for the *D. melanogaster* reference gene for a given isoform and run it using *tblastn* against their target *Drosophila* species genome assembly on the NCBI BLAST server ( https://blast.ncbi.nlm.nih.gov/Blast.cgi ; Altschul et al., 1990) to identify potential orthologs. To validate the potential ortholog, students compare the local genomic neighborhood of their potential ortholog with the genomic neighborhood of their reference gene in *D. melanogaster* . This local synteny analysis includes at minimum the two upstream and downstream genes relative to their putative ortholog. They also explore other sets of genomic evidence using multiple alignment tracks in the Genome Browser, including BLAT alignments of RefSeq Genes, Spaln alignment of *D. melanogaster* proteins, multiple gene prediction tracks (e.g., GeMoMa, Geneid, Augustus), and modENCODE RNA-Seq from the target species. Detailed explanation of how these lines of genomic evidenced are leveraged by students in gene model development are described in Rele et al. (2023). Genomic structure information (e.g., CDSs, intron-exon number and boundaries, number of isoforms) for the *D. melanogaster* reference gene is retrieved through the Gene Record Finder ( https://gander.wustl.edu/~wilson/dmelgenerecord/index.html ; Rele et al *.,* 2023). Approximate splice sites within the target gene are determined using *tblastn* using the CDSs from the *D. melanogaste* r reference gene. Coordinates of CDSs are then refined by examining aligned modENCODE RNA-Seq data, and by applying paradigms of molecular biology such as identifying canonical splice site sequences and ensuring the maintenance of an open reading frame across hypothesized splice sites. Students then confirm the biological validity of their target gene model using the Gene Model Checker ( https://gander.wustl.edu/~wilson/dmelgenerecord/index.html ; Rele et al., 2023), which compares the structure and translated sequence from their hypothesized target gene model against the *D. melanogaster* reference gene model. At least two independent models for a gene are generated by students under mentorship of their faculty course instructors. Those models are then reconciled by a third independent researcher mentored by the project leaders to produce the final model. Note: comparison of 5' and 3' UTR sequence information is not included in this GEP CURE protocol.

## Data Availability

Description: A GFF, FASTA, and PEP of the model. Resource Type: Model. DOI: https://doi.org/10.22002/dy8va-kmb97
